# Longitudinal adrenal gland measurements and growth trajectories as risk markers for late preterm delivery

**DOI:** 10.1186/s12884-020-03255-6

**Published:** 2020-09-29

**Authors:** Janina Goletzke, Mirja Pagenkemper, Christian Wiessner, Franziska Rüber, Petra Arck, Kurt Hecher, Anke Diemert

**Affiliations:** 1 Department of Obstetrics and Fetal Medicine, University Medical-Centre Hamburg Eppendorf, Martinistrasse 52, 20246 Hamburg, Germany; 2 Institute of Medical Biometry and Epidemiology, University Medical-Centre Hamburg Eppendorf, Hamburg, Germany

**Keywords:** Preterm labour, Adrenal gland, Fetal zone, Fetal adrenal gland, Preterm birth, HPA-axis, Fetal ultrasound

## Abstract

**Background:**

The fetal adrenal gland receives rising awareness as a predictor of spontaneous preterm birth. We hereby provide longitudinal growth assessments of the fetal adrenal gland in a low risk population with an additional focus on trajectories in fetuses born preterm.

**Methods:**

Fetal adrenal gland was assessed via transabdominal ultrasound at gestational weeks (gw) 24–26, 28–30, and 34–36 in a low-risk pregnancy cohort. Longitudinal trajectories of the total gland and the mark (so called fetal zone) as well as ratio of fetal zone width/ total widths (w/W) were analyzed using repeated ANOVA analyses. To compare trajectories of the ratio w/W for preterm and term fetuses respectively, as well as women with and without clinical signs of preterm labor, the propensity score method was applied.

**Results:**

Fetal zone width increased over the course of pregnancy (*p* < 0.0001), while the ratio w/W decreased (*p* < 0.0001) (*n* = 327). Comparing the trajectories of the ratio w/W in fetuses born preterm (*n* = 11) with propensity-score matched term born fetuses (*n* = 22), a decrease between gw 24–26 and 28–30 was observed in both groups, which continued to decrease for the term born fetuses. However, in preterm born fetuses, the ratio increased above the term born values at gw 34–36.

**Conclusion:**

Our study provides for the first time longitudinal growth data on the fetal adrenal gland and supports the hypothesis that fetal zone enlargement is associated with preterm birth which could play an important role in risk-prediction.

## Background

Preterm delivery (< 37 weeks) still remains the leading cause of perinatal morbidity and mortality in developed countries of which the majority of cases are late preterm infants (occurring between 34 and 37 weeks) [1]*.* Although the detailed mechanisms of the parturition process and preterm birth remains elusive, intervention strategies have recently been introduced [2–4]. Unfortunately, we still lack accurate predictors of preterm birth [5].

It is well known, that the fetal adrenal gland plays an important role in parturition [6]*.* As part of the fetal adrenocortical axis it is involved in the general fetal stress response [7–10].

A number of cross-sectional acquired reference ranges have been proposed for total adrenal gland size [11–13] and the distinction between the adrenal mark (called fetal zone) and the adrenal cortex [14, 15]*.* However, none of them was based on repeated longitudinal assessments or individual growth trajectories of the fetal adrenal gland.

Against this background previous studies investigated the suitability/usefulness of various ultrasound measurements of the adrenal gland for predicting preterm birth primarily in women with signs of preterm labour or premature rupture of membranes (PROM).

Looking at the volume of the whole adrenal gland, Turan and colleagues found larger volumes in women who gave birth within the next 5 days compared to those who did not [16] and described that predominantly the fetal zone increases in size. The ratio between the fetal zone and the whole adrenal gland could accurately distinguish between patients with symptoms of preterm birth who delivered within the next week and those who did not with high sensitivity and specificity [15]*.* The groups of Santipap et al. as well as Agarwal reported that in patients with either threatened preterm labour or preterm labour an enlarged adrenal gland in combination with high levels of placental alpha macroglobulin-1 or cervical elastography respectively was effective in predicting preterm delivery within 7 days [17, 18]. The adrenal gland ratio was reported to be independent of gestational age [9] and is therefore particular useful for screening.

In contrast, in a large multicenter observational cohort study of asymptomatic nulliparous women, cross-sectional adrenal gland measurements failed to identify preterm delivery [14]*.*

The aim of this study was to provide longitudinal assessments of the fetal adrenal gland in a low risk population. Additionally, taking advantage of our carefully and repeatedly collected data, we aim to test the hypothesis of fetal adrenal zone enlargement prior to (preterm) birth. In order to do so, in a secondary analysis, fetal adrenal gland trajectories were assessed separately for a small subsample of preterm and term born neonates as well as women with and without preterm labor.

## Methods

### Study design and population

Since 2011 women were recruited via resident gynecologists to the prospective pregnancy cohort PRINCE (PRENATAL DETERMINANTS OF CHILDREN’S HEALTH) at the University Medical Center Hamburg-Eppendorf. Women of legal age with a viable singleton pregnancy confirmed by first trimester ultrasound at gestational age of 12–14 weeks were invited to three antenatal visits, scheduled once per trimester (gestational week 12 to 14, 24 to 26, and 34 to 36) after obtaining written consent. Multiples or pregnancies conceived after assisted reproductive technologies (ART), maternal chronic infections (HIV, hepatitis B/C) and known substance abuse were exclusion criteria. The study protocol was approved by the ethics committee of the Hamburg Chamber of Physicians.

### Ultrasound measurements

Ultrasound measurements were performed using a Voluson E8 (General Electric; GE), equipped with a transabdominal 3–5 MHz transducer (RAB 6D, GE). Measurements were carried out by two fully trained gynecologists (M.P. and F.R.) with certified advanced ultrasound expertise. In each case a routine standard sonographic study, including fetal biometry and anomaly screening was performed first. Estimated fetal weight was obtained using the Hadlock IV formula [19].

The fetal adrenal gland was assessed for the first time during the study visit at gestational weeks 24. Measurements of length, width and depth of the total fetal adrenal gland as well as of the fetal zone, were performed in triplicate if possible, and the mean of these measurements was calculated. We measured the length and width in a 2-dimensional transverse plane with the spine in lateral position. Furthermore, a ratio was calculated relating the zone width to the total adrenal gland width, measured in the same plane (will be hereafter referred to as ‘ratio w/W’). Additionally, we acquired a 3 D Volume and used this for assessment of the adrenal gland depth in the sagittal plane.

**Fig. 1:**
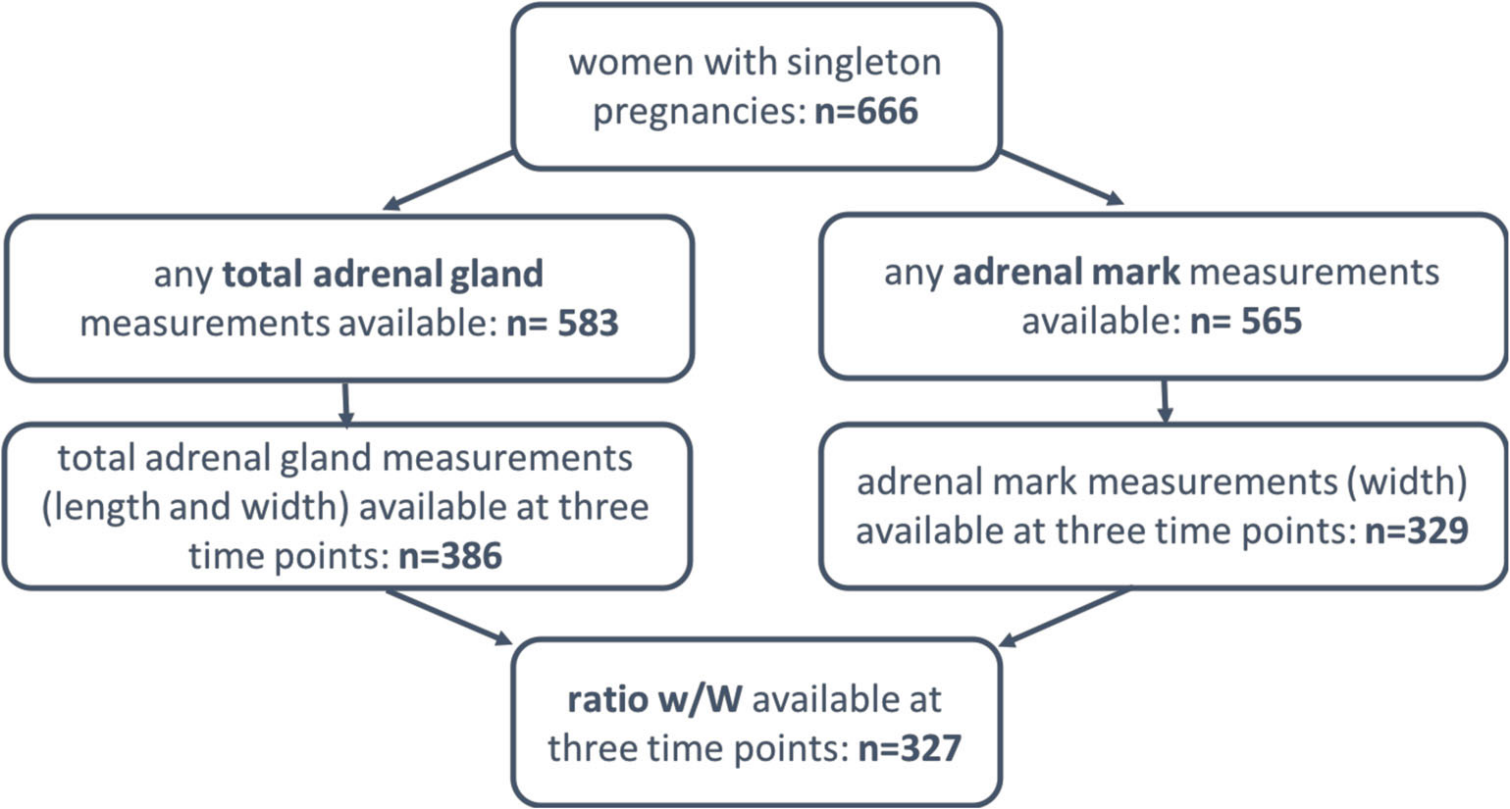
Flow chart of available adrenal gland assessments; three measurement time points refer to gestational weeks 24–36, 28–30, and 34–36. ratio w/W = ratio fetal zone width / total adrenal gland width

If available, adrenal gland volume was calculated additionally as suggested by Turan et al. [15] using the ellipsoid formula (length*width*depth*0.523). As shown in Fig. 1, fetal adrenal gland measurements could not be performed in all study objects at every study visit due to e.g., the fetal position, movement or unfavorable maternal body composition.

### Assessment of relevant covariates

Upon inclusion we collected data on the sociodemographic background, general health and chronic diseases (such as hypertension, hypo−/hyperthyreosis, asthma or kidney disease) from our participants. At each each study visit, maternal anthropometry was assessed by measuring to the nearest 0.5 cm or 0.1 kg respectively (light clothing, no shoes) in a standardized way.

### Statistical analyses

To quantify the concordance between two independent observers, we assessed inter-observer reliability using intra-class correlations (ICCs) for the assessment of total adrenal length and width as well as fetal zone width. Additionally, to assess the range of variation as well as systemic measurement errors graphically, Bland-Altman diagrams were used. To present the study sample characteristics, mean (SD) as well as total number (percentages) were presented for relevant maternal and obstetric characteristics. To assess longitudinal development of the total adrenal gland, median values of length, width, and depth measurements as well as the total volume were assessed at all three relevant study visits. Likewise, longitudinal trajectories of the median fetal zone width as well as ratio w/W were presented and analyzed using repeated measurements ANOVA.

As enlargement of the fetal zone (i.e. an increase of the ratio w/W) is discussed as a potential predictor for preterm birth [16] we aimed to additionally compare trajectories of the ratio w/W for preterm and term born neonates as well as women with preterm labor and without any complication. Due to the low prevalence of preterm birth / pregnancy complications in our healthy study cohort, we used a propensity score method for these comparisons which proved to be appropriate for a low number of events per confounder [20, 21]. Propensity scores were determined using logistic regression models in all participants with ratio w/W measures and information on gestational week at delivery or pregnancy complications, respectively. Propensity score matching (1:2) was performed based on estimated fetal weight, fetal gender, parity, maternal age and maternal first trimester BMI. Calipers of width equal to 0.4 standard deviations of the logit of the propensity score, respectively, were calculated separately per subgroup. Descriptive statistics indicated that measured confounders were well balanced between both groups after matching (**Supp.** Tab. 1) [22]. Matched groups were compared using paired t-tests to account for the clustered structure [20]. All analyses were carried out by using SAS software (version 9.4; SAS Institute) and were performed with a significance level at *p* < 0.05.

## Results

Because of the open cohort design, complete data was not present for all participants. At the time of this analysis, data of 666 women with singleton pregnancies was available. Any measurement of the total adrenal gland and the fetal zone was available for 583 and 565 participants respectively. Complete data (length and width of the adrenal gland) on all three relevant study visits (gw 24–26, 28–30, and 34–36) was available for 386 and 329 participating women respectively. Total volume, because it requires depth measurements, was only calculated for a small fraction (*n* = 30) at all three time points. (Fig. 1).

Regarding data quality, intra-class correlation coefficients (ICC) were calculated in a subgroup of samples and showed excellent agreement for all adrenal gland assessments between the two sonographers (Table 1). Bland-Altman diagrams indicated furthermore a range of variation of +/− 1 mm and no systematic measurement bias (data not shown).

**Table 1:** Assessment of inter-observer reliability using intra-class correlation coefficients (ICC)

Ultrasound parameter	ICC^a^	***p***-value
total adrenal gland length	0.936 (0.791, 0.981)	< 0.001
total adrenal gland width	0.831 (0.472, 0.947)	0.002
fetal zone width	0.840 (0.466, 0.952)	0.002

^a^Intra-class correlation coefficients (ICC) with 95% confidence intervals in parenthesis

The characteristics of the study population are shown in Table 2: Mean maternal age at birth was 31.4 years and mean maternal first trimester BMI was 24.4 kg/m^2^. The PRINCE cohort is characterized by a high socioeconomic status as shown by the high percentage of women with a university degree (49%). 37% of the participating women had already one or more children and 30% of the women experienced a complication in their present pregnancy (hypertensive disorder, gestational diabetes, bleeding, threatened preterm labour, infections). Preterm birth occurred in 32 out of 578 cases (5.5%).

**Table 2:** Characteristics of the study population (*n* = 583)

	n	mean (SD)
**maternal characteristics**		
Age	583	31.38 (3.62)
first trimester BMI	583	24.40 (4.01)
education; n (%)	564	
main or middle school		121 (21.5%)
high school graduation		167 (29.6%)
university degree		276 (48.9%)
pre-existing medical condition; n (%)	565	134 (23.7%)
parity ≥1; n (%)	581	213 (36.7%)
**obstetric characteristics**		
Birthweight	578	3476 (486)
gestational week	578	40.0 (1.6)
preterm-born neonates (<gw 37 + 0), n (%)	578	32 (5.5%)
pregnancy complications, n (%)	572	172 (30.1%)
preterm labor		34 (5.9%)
others (i.e. GDM, pregnancy induced hypertension)		138 (24.1%)

**Fig. 2:**
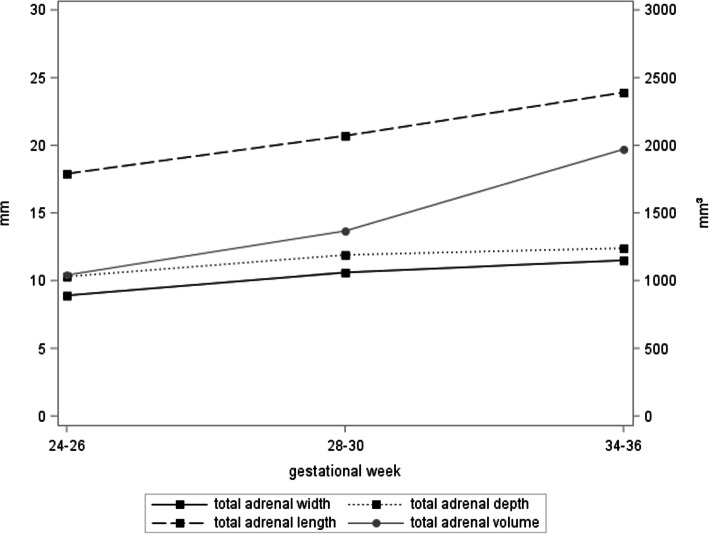
Development of total adrenal length and width measures (*n* = 386) and total adrenal depth and volume measures (*n* = 30) over the course of pregnancy. Total adrenal volume was calculated using the ellipsoid formula (length*width*depth*0.523)

**Fig. 3:**
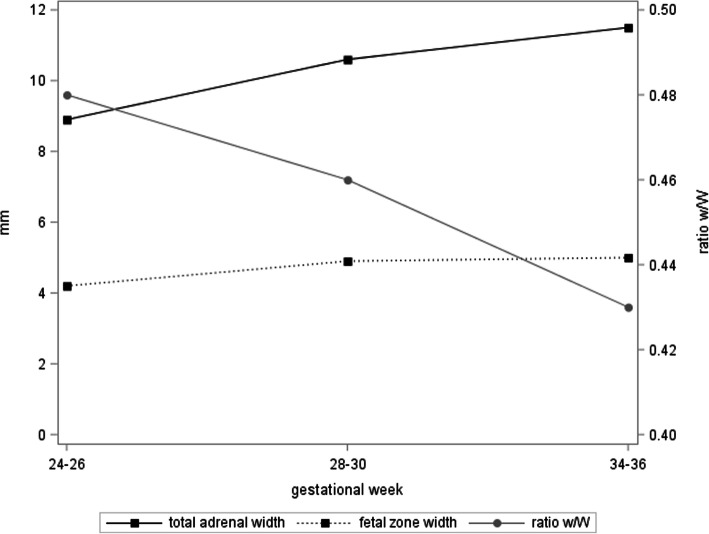
Development of total adrenal width (*n* = 386), fetal zone width (*n* = 329) and the ratio of fetal zone width / total adrenal gland width (w/W) (*n* = 327) over the course of pregnancy

Figure 2 presents the longitudinal trajectories for total adrenal gland measures for those participants with available measures at all three time points. With the exception of adrenal depth, a statistically significant increase of these measures was observed over the course of pregnancy (*p* < 0.0001 for adrenal length, width and volume). Likewise, the fetal zone width increased over the course of pregnancy (*p* < 0.0001), while the ratio w/W decreased (*p* < 0.0001), indicating a comparatively larger increase of the total adrenal gland compared to the fetal zone (Fig. 3).

Regarding the relation between estimated fetal weight and adrenal gland measures, a positive association was observed for total adrenal width as well as total adrenal area at all three study visits with an explained variance between 4 and 13%. Likewise, a positive association was observed for total adrenal volume at gestational weeks 24–26 and 28–30 (explained variance 15.1–20.3%). In contrast, estimated fetal weight explained only 0.01–1.0% of the variance in the ratio w/W (Table 3). Because of the observed fetal weight-independency of the ratio w/W, we subsequently focused our analyses on this measure.

**Table 3:** Cross-sectional association between estimated fetal weight and selected fetal adrenal gland measures

independent variable: estimated fetal weight				
	n	ß (SE)	R^**2**^	p
	**dependent variable:** total adrenal gland width (mm)			
**gw 24–26**	386	0.0064 (0.0012)	0.071	< 0.0001
**gw 28–30**	386	0.0025 (0.0006)	0.041	< 0.0001
**gw 34–36**	386	0.0020 (0.0003)	0.078	< 0.0001
	**dependent variable:** total adrenal gland area (mm^2^)			
**gw 24–26**	386	0.2400 (0.0311)	0.134	< 0.0001
**gw 28–30**	386	0.0780 (0.0196)	0.040	< 0.0001
**gw 34–36**	386	0.0778 (0.0120)	0.098	< 0.0001
	**dependent variable:** total adrenal volume (mm^3^) (length*width*depth*0.523 (ellipsoid formula))			
**gw 24–26**	30	1.1984 (0.7434)	0.203	0.0125
**gw 28–30**	30	1.2931 (0.5791)	0.151	0.0337
**gw 34–36**	30	0.4589 (0.3754)	0.051	0.2318
	**dependent variable:** ratio w/W			
**gw 24–26**	327	0.00001 (0.00007)	0.0001	0.8389
**gw 28–30**	327	−0.00006 (0.00005)	0.010	0.1736
**gw 34–36**	327	0.00001 (0.00002)	0.001	0.5562

*acc. to Hadlock (gramm)

**Fig. 4:**
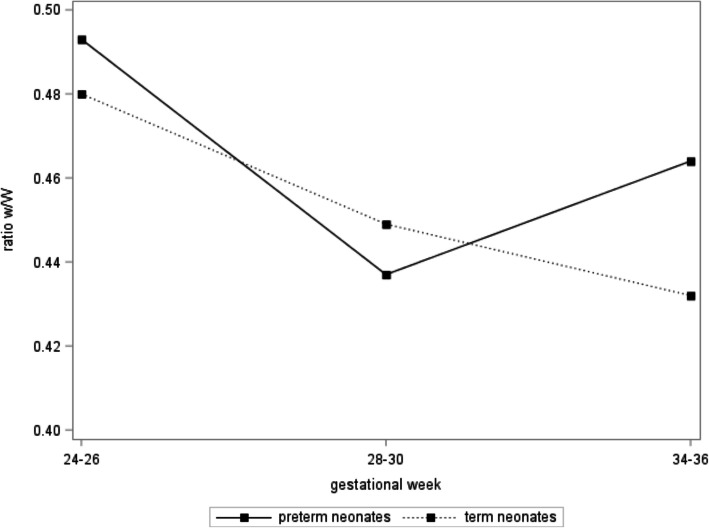
Ratio fetal zone width / total adrenal gland width (w/W) over the course of pregnancy, presented separately for those neonates born term and preterm. Preterm and term born neonates were propensity score matched (1:2) (*n* = 11 preterm and *n* = 22 term born neonates)

**Fig. 5:**
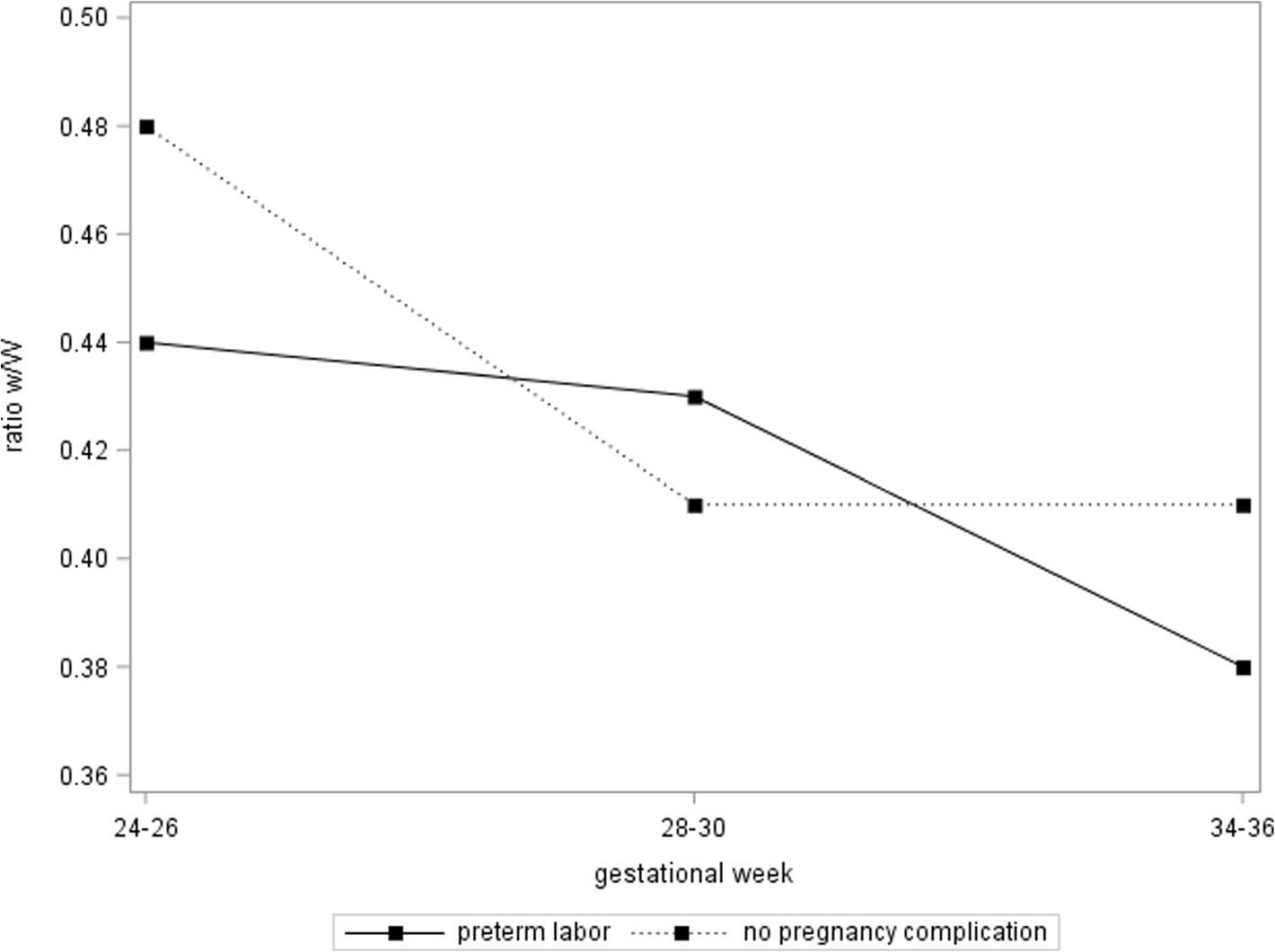
Ratio fetal zone width / total adrenal gland width (w/W) over the course of pregnancy, presented separately for those women with preterm labor and those without any pregnancy complication. These women were propensity score matched (1:2) (*n* = 11 women with preterm labor and *n* = 22 with no complication)

Figure 4 presents ratio w/W trajectories for propensity score-matched pairs of preterm and term born neonates. The ratio w/W in term born neonates decreases as observed for all participants (*p* < 0.0001). However, a different pattern emerges for preterm neonates: While the ratio w/W decreases between gestational weeks 24–26 and 28–30, it increases above the term born values at gestational weeks 34–36 (p for difference = 0.7, 0.6, and 0.2 for preterm vs. term at gestational weeks 24–26, 28–30 and 34–36, respectively). Different trajectories were observed when comparing propensity score-matched women with preterm labor and those without any pregnancy complication: In contrast to the observed increase for preterm born neonates, for both groups (preterm labor and no complication) decreasing trajectories of the ratio w/W were observed (Fig. 5).

## Discussion

This study provides for the first-time longitudinal growth data for the adrenal gland and the fetal zone in pregnancies that resulted in term or preterm birth in an otherwise healthy study population. Previous normograms for fetal adrenal growth were derived from cross-sectional studies. Our study design allowed us to investigate intra-individual growth trajectories for the fetal adrenal gland.

In line with the previous observations our study confirms that enlargement of the fetal zone detected by ultrasound up to 3 weeks before birth is associated with preterm birth irrespective of other clinical signs of preterm labor. While our study was not designed to assess predictors of preterm birth, we aimed to test the hypothesis of fetal zone enlargement prior to preterm birth in a healthy cohort where repeated assessments of the fetal adrenal gland were available.

There are still various methods to measure the fetal adrenal gland via transabdominal ultrasound [12, 14, 15, 23, 24]*.* We opted to focus on two-dimensional adrenal gland width and length measurements due to their superiority in regard to image quality, feasibility, visibility of the borders, and reliability in accordance with other colleagues [9, 10, 14, 15]*.* Adrenal width and length measurements were carried out in the transverse plane allowing us to evaluate at the same time the physiologically relevant fetal zone in relation to the total adrenal gland without shadowing from the ribs (**Supp.** Fig. 1**)**.

We observed an association between adrenal measures and fetal size. With the exception of adrenal depth, a statistically significant increase of adrenal measures was observed over the course of pregnancy (*p* < 0.0001 for adrenal length, width and volume). In concordance with the study by Iijima et al., in our study the influence of fetal size on adrenal gland measurements seems to decrease as pregnancy progressed and the variance explained by fetal size is lowest in the third trimester of pregnancy. Other aspects such as fetal distress may instead account for the observed variations [10]. For the ratio fetal zone width / total adrenal gland width however we observed decreasing values over the course of pregnancies, indicating a comparatively larger increase of the total adrenal gland compared to the fetal zone. The ratio fetal zone width / total adrenal gland width provides us therefore with a fetal weight-independent parameter and we hence focused our subsequent analyses of preterm birth prediction on this marker [9, 10, 15]*.*

Comparing the trajectories of this ratio in fetuses born preterm with propensity-score matched term born fetuses, we observed in both groups a decrease between gestational weeks 24–26 and 28–32, which continued to decrease for the term born fetuses. However, in those fetuses destined to be born preterm, the ratio increased above the term born values at gestational weeks 34–36. Trajectories therefore clearly distinguished between the fetuses destined to be born preterm and the ones that go on to be born at term.

In addition, we compared propensity score-matched women with preterm labor and those without any symptoms of preterm birth or other pregnancy complication. In contrast to the observed increase for preterm born neonates, for both groups (with and without symptoms of preterm labor) decreasing trajectories of the ratio w/W were observed. To our surprise, when the occurrence of preterm labor (yes/no) was further added to the multivariate model, the significant effect of the w/W ratio at gestational week 28–30 remained (*p* = 0.0486) and the explained variance in gestational age increased only from 30 to 31%. This implicates that using the w/W ratio to screen for preterm birth also picks up the cases without any clinical warning signs such as symptoms of preterm birth and is indeed applicable both in a high-risk and in a low risk population. This result clearly distinguishes our work from previous publications that mostly evaluated patients already identified to be at risk for preterm labor by their clinical signs. However, identifying the cases not picked up by symptoms would be novel and beneficial.

Of note, in a large cross-sectional multicenter observational cohort study of asymptomatic nulliparous women (*n* = 1723) adrenal gland measurements failed to identify pregnancies delivering preterm (< 37 0/7 weeks) [14], although in previous publications Turan et al. have shown the opposite in symptomatic women before [15, 16]*.* There are several possible explanations for these discrepant findings.

The time interval between adrenal gland measurement and delivery seems of importance [14, 15] for its predictive value. The hypothalamic pituitary axis has the capacity to immediately react to external stressors and therefore it is conceivable that changes in the adrenal gland occur over a short period of time. Turan et al. themselves showed that the sensitivity in predicting preterm birth by adrenal gland enlargement was higher the shorter the remaining interval to birth was (from 94% sensitivity 24 h before to 61% sensitivity 2 weeks before birth respectively) [16]. Whereas the preterm delivery took place 5 days after the last adrenal gland measurement in their original publication of 2011 [15], in the subsequent prospective trial of only nulliparous women [14] this interval was much longer, on average 55 days. In our (late) preterm-group the scan took place less than 3 weeks before birth, where the fetal zone enlargement was evident (it should be noted that we had just 11 cases in this group). Of note, we included only cases with adrenal gland measurements at all three time points as we aimed to present longitudinal trajectories in a low-risk population and not preterm birth. We excluded by definition patients that resulted in preterm birth before participating in our last study visit at a minimum of 34 + 0 gw but none of these delivered as early preterms (*n* = 3 neonates were born < 34 gestational weeks, i.e. at gestational week 32 or 33). The parent study PRINCE resulted in a cohort of parturients of higher socio-economic status with a low risk for preterm birth and other severe pregnancy complications. To address the low prematurity rate in our cohort with only few women with preterm labor and complete data on all three study visits, we used the method of propensity score matching for a quasi-randomized analysis, where possible confounders were equally distributed.

We assume that the adrenal gland measurement seems to be of value as an acute marker of spontaneous preterm birth rather than an early marker, such as cervical length [5]. Cervical measurement is much stronger in predicting preterm birth in general than the time interval to delivery or late preterm delivery. Unfortunately, due to our study design, we did not have cervical measurements on all of our preterm deliveries in order to compare both prediction models. However others have compared both and shown it to be at least as effective [18] if not more superior [15]. The lack of this comparison is a clear limitation of our study and hampers final conclusions. Moreover, it has to be noted that preterm birth is a combined outcome of different antecedent pathways such as inflammation, myometrial stretch, activation of the maternal-fetal Hypothalamic Pituitary- Adrenal (HPA) axis and decidual changes [25]. It is conceivable that changes in the fetal zone may not occur if the pathway to preterm parturition is not through activation of the maternal-fetal HPA. As a consequence, screening for preterm birth should include various complementing assessments such as cervical length and fetal zone measurements. A further limitation of our study is the limited data on pregnancy complications, for which the prevalence was overall small, and occurrences were heterogenous, preventing us from detailed analyses. We choose to focus on preterm labor as a pregnancy complication specifically as it was the best comparator for preterm birth. However, it should be noted that additional analyses showed similar trajectories for those women experiencing pregnancy complications other than preterm labor.

We hereby present for the first-time longitudinal growth-data on the fetal adrenal gland and observe that enlargement of adrenal gland is predictive for preterm birth irrespective from clinical signs of preterm labor according to our secondary data-analysis in a small subsample of the present cohort. This observation warrants further confirmation in a larger cohort specifically designed to address pre-term birth.

## Conclusion

Our study provides for the first time longitudinal growth data on the fetal adrenal gland and supports the hypothesis that fetal zone enlargement is associated with preterm birth which could play an important role in risk-prediction.

## Supplementary information

**Additional file 1: Table S1.** Distribution of the variables considered for propensity score calculation.

**Additional file 2: Figure S1.** Ultrasound measurement of the fetal adrenal gland in a 2-dimensional transverse plane with the spine in lower left quadrant (star). Depicted is the spine, the fetal zone gland zone width (continuous arrow) and the total adrenal gland width (dashed arrow) at gestational age 28 weeks.

## Data Availability

The datasets used and/or analysed during the current study are available from the corresponding author on reasonable request.

## Abbreviations

gw: Gestational week
w/W: Fetal zone width/total adrenal gland width
n: Number
PROM: Premature Rupture of Membranes
PRINCE: Prenatal Identification of Children’s health
HIV: Human Immundeficiency Virus
ART: Assisted Reproductive Technologies
GE: General electrics
MHz: Mega Hertz
3D: Three Dimensional
ICC: Intra-Class Correlation
BMI: Body Mass Index
mm: Millimeter
HPA: Hypothalamic-Pituitary-Adrenal
