# Supplementary material for: Longitudinal adrenal gland measurements and growth trajectories as risk markers for late preterm delivery
Source: BMC Pregnancy Childbirth. 2020 Sep 29;20:570. doi: 10.1186/s12884-020-03255-6 (PMC7526396; doi:10.1186/s12884-020-03255-6)
Supplement: Supplementary file 1 — Additional file 1: Table S1. Distribution of the variables considered for propensity score calculation. [file 12884_2020_3255_MOESM1_ESM.docx]

**Supplemental Table 1:** Distribution of the variables considered for propensity score calculation

| **Preterm vs. term born neonates:** | | | |
| --- | --- | --- | --- |
|  | **preterm neonates**  n=11 | **term neonates**  n=22 | **p**  for difference |
| estimated fetal weight gw 23-25 (gramm) | 676 (102) | 662 (73) | 0.5 |
| fetal gender (girl), n (%) | 7 (63.6%) | 14 (63.6%) | 1.0 |
| parity, ≥1, n (%) | 6 (54.6%) | 16 (48.5%) | 0.3 |
| maternal age (years) | 33.9 (3.2) | 34.6 (2.6) | 0.3 |
| maternal first trimester BMI (kg/m^2^) | 25.6 (3.6) | 24.2 (3.9) | 0.3 |
| **Women with preterm labor vs. no pregnancy complication** | | | |
|  | **preterm labor**  n=11 | **no complication**  n=22 | **p**  for difference |
| estimated fetal weight gw 23-25 (gramm) | 631 (48) | 609 (88) | 0.3 |
| fetal gender (girl), n (%) | 6 (54.6%) | 12 (54.6%) | 1.0 |
| parity, ≥1, n (%) | 2 (18.2%) | 6 (27.3%) | 0.6 |
| maternal age (years) | 30.8 (4.1) | 30.7 (3.8) | 0.8 |
| maternal first trimester BMI (kg/m^2^) | 22.8 (2.1) | 22.6 (3.0) | 0.8 |
