# Supplementary material for: Longitudinal adrenal gland measurements and growth trajectories as risk markers for late preterm delivery
Source: BMC Pregnancy Childbirth. 2020 Sep 29;20:570. doi: 10.1186/s12884-020-03255-6 (PMC7526396; doi:10.1186/s12884-020-03255-6)
Supplement: Supplementary file 2 — Additional file 2: Figure S1. Ultrasound measurement of the fetal adrenal gland in a 2-dimensional transverse plane with the spine in lower left quadrant (star). Depicted is the spine, the fetal zone gland zone width (continuous arrow) and the total adrenal gland width (dashed arrow) at gestational age 28 weeks. [file 12884_2020_3255_MOESM2_ESM.docx]

**Supplemental Figure 1:** Ultrasound measurement of the fetal adrenal gland in a 2-dimensional transverse plane with the spine in lower left quadrant (star). Depicted is the spine, the fetal zone gland zone width (continuous arrow) and the total adrenal gland width (dashed arrow) at gestational age 28 weeks.
